# Type I Interferon Supports Inducible Nitric Oxide Synthase in Murine Hepatoma Cells and Hepatocytes and during Experimental Acetaminophen-Induced Liver Damage

**DOI:** 10.3389/fimmu.2017.00890

**Published:** 2017-07-31

**Authors:** Malte Bachmann, Zoe Waibler, Thomas Pleli, Josef Pfeilschifter, Heiko Mühl

**Affiliations:** ^1^Pharmazentrum Frankfurt/ZAFES, University Hospital, Goethe University Frankfurt, Frankfurt am Main, Germany; ^2^Junior Research Group “Novel Vaccination Strategies Early Immune Responses”, Paul-Ehrlich-Institut, Langen, Germany; ^3^Department of Medicine I, University Hospital, Goethe University Frankfurt, Frankfurt am Main, Germany

**Keywords:** type I interferon, inducible nitric oxide synthase, signal transducer and activator of transcription-1, acetaminophen, liver damage

## Abstract

Cytokine regulation of high-output nitric oxide (NO) derived from inducible NO synthase (iNOS) is critically involved in inflammation biology and host defense. Herein, we set out to characterize the role of type I interferon (IFN) as potential regulator of hepatic iNOS *in vitro* and *in vivo*. In this regard, we identified in murine Hepa1-6 hepatoma cells a potent synergism between pro-inflammatory interleukin-β/tumor necrosis factor-α and immunoregulatory IFNβ as detected by analysis of iNOS expression and nitrite release. Upregulation of iNOS by IFNβ coincided with enhanced binding of signal transducer and activator of transcription-1 to a regulatory region at the murine iNOS promoter known to support target gene expression in response to this signaling pathway. Synergistic iNOS induction under the influence of IFNβ was confirmed in alternate murine Hepa56.1D hepatoma cells and primary hepatocytes. To assess iNOS regulation by type I IFN *in vivo*, murine acetaminophen (APAP)-induced sterile liver inflammation was investigated. In this model of acute liver injury, excessive necroinflammation drives iNOS expression in diverse liver cell types, among others hepatocytes. Herein, we demonstrate impaired iNOS expression in type I IFN receptor-deficient mice which associated with diminished APAP-induced liver damage. Data presented indicate a vital role of type I IFN within the inflamed liver for fine-tuning pathological processes such as overt iNOS expression.

## Introduction

High-output nitric oxide (NO) production achieved by inducible NO synthase (iNOS) is key to efficient innate host defense but also involved in pathological inflammation at diverse organs including the liver (1–4). There, iNOS is detectable in several cell types including Kupffer cells and hepatocytes. Accordingly, expression and biological activity of iNOS has been related to the pathogenesis of acute and chronic liver diseases, among others drug-induced liver injury, non-alcoholic fatty liver disease/steatohepatitis, alcoholic liver disease, liver fibrosis, viral hepatitis, and carcinogenesis (3, 4). Especially under conditions of glutathione depletion, hepatotoxicity by xenobiotics is frequently mediated at least partly by the highly reactive NO metabolite peroxynitrite (5).

Regulation of iNOS expression and activity occurs foremost on the level of gene transcription with pathways activating nuclear factor (NF)-κB and signal transducer and activator of transcription (STAT)-1 being of particular importance. Depending on the biochemical and cellular context STAT1 is able to support iNOS expression as STAT1 homodimer or as part of a protein complex together with STAT2 and IRF9 known as interferon (IFN)-stimulated gene factor (ISGF)-3 (1, 2, 6–9). Particularly in non-leukocytic cells such as renal mesangial cells and hepatocytes, iNOS mRNA is amplified by NO-driven feed-forward mechanisms (10, 11). Whereas a crucial role for IFNγ concerning iNOS induction is established in diverse cell types including hepatocytes (12), information on the role of immunoregulatory type I IFN (including IFNα/β) as cofactor for induction of hepatocyte iNOS is currently lacking. Of note, IFNα/β is reported to efficiently drive monocyte/macrophage-derived iNOS in humans and mice (9, 13–15). Moreover, in combination with interleukin (IL)-22, IFNα upregulates expression of iNOS in human DLD1 colon carcinoma cells (16). Herein, we set out to further characterize *in vitro* and *in vivo* the role of type I IFN for murine hepatic iNOS regulation by using the cellular model of IFNβ-stimulated hepatoma cells (Hepa1-6, Hepa56.1D) and hepatocytes and by investigating murine acetaminophen (paracetamol, APAP)-induced sterile liver inflammation in the context type I IFN receptor-deficient mice.

## Materials and Methods

### Reagents

Human IL-1β and murine tumor necrosis factor (TNF)-α were from Peprotech, Inc. (Frankfurt, Germany). Murine IFNβ was purchased from PBL (New York, USA) and APAP from Sigma-Aldrich (Taufkirchen, Germany). Inhibitor-κB kinase (IKK)-VII inhibitor was from Calbiochem/Merck Millipore (Darmstadt, Germany).

### Cultivation of Murine Hepa 1-6 and Hepa56.1D Cells

Hepa1-6 cells (LGC Standards, Wesel, Germany) and Hepa56.1D (CLS GmbH, Eppenheim, Germany) were maintained in DMEM supplemented with 100 U/ml penicillin, 100 µg/ml streptomycin, and 10% heat-inactivated FCS (Thermo Fisher Scientific, Langenselbold, Germany). For experiments, cells were seeded on six-well polystyrene plates (Greiner, Frickenhausen, Germany) in the aforementioned medium.

### Isolation of Primary Murine Hepatocytes

C57Bl/6 mice were sacrificed and obtained livers were perfused *post mortem*. The isolation procedure was adapted from Godoy et al. (17). Briefly, perfusion was performed with 37°C warm HBSS without Ca^2+^ and Mg^2+^ (supplemented with 15 mM HEPES, 2.5 mM EGTA, 1 g/l glucose, 100 U/ml penicillin, and 100 µg/ml streptomycin) using a roller pump (10 ml/min) for 10 min. Thereafter, the liver was perfused with HBSS with Ca^2+^ and Mg^2+^ [supplemented with 15 mM HEPES, 5 mM CaCl_2_, 0.13 mg/ml collagenase IV (Sigma-Aldrich, Darmstadt, Germany), 100 U/ml penicillin, and 100 µg/ml streptomycin] for additional 10 min. The liver was carefully removed from the abdominal cavity, placed in a Petri dish on ice in DMEM (supplemented with 10% FCS, 100 U/ml penicillin, and 100 µg/ml streptomycin) and opened with a forceps. Liver cells were resuspended and put over a 100 µm cell strainer (Becton Dickinson, Heidelberg, Germany). After two rounds of centrifugation (5 min at 50 g and 4°C) and resuspension, cell viability was determined by trypan blue dye exclusion, and cells were seeded in DMEM (supplemented with 10% FCS, 100 U/ml penicillin, and 100 µg/ml streptomycin) on collagen G-coated plates (Biochrom, Berlin, Germany). Adherent hepatocytes were washed after 4 h with PBS and fresh Williams Medium E [supplemented with 10% FCS, 2 mM l-Alanyl-l-Glutamin (Biochrom), 2 ng/ml insulin, 100 ng/ml dexamethasone, 100 U/ml penicillin, and 100 µg/ml streptomycin] was added. Stimulation with recombinant cytokines for analysis of iNOS expression was performed 16 h thereafter. Dexamethasone was not removed from standard supplemented Williams Medium E because this glucocorticoid is known to inhibit hepatocyte apoptosis (18, 19) thereby supporting viability. This protocol was adhered to despite the prospect that dexamethasone at this concentration is capable of reducing NF-κB thus partly affecting hepatocyte iNOS (20).

### Detection of CXCL9, IFNα, IFNβ, iNOS, MIP2, and STAT1 mRNA

Total RNA isolation was performed as described (16). Briefly, RNA isolated by Tri-Reagent (Sigma-Aldrich) was transcribed using random hexameric primers (Qiagen, Hilden, Germany) and Moloney virus reverse transcriptase (Thermo Fisher Scientific). During realtime PCR, changes in fluorescence are caused by the Taq polymerase degrading a probe containing a fluorescent dye (GAPDH: VIC; all others: FAM). Pre-developed reagents (Thermo Fisher Scientific): GAPDH (4352339E), CXCL9 (Mm00434946_m1), IFNα2 (Mm00833961_s1), IFNα4 (Mm00833969_s1), IFNα5 (Mm00833976_s1), IFNβ (Mm00439552_s1), STAT1 (Mm00439531_m1), iNOS (Mm00440502_m1), and MIP2 (Mm00436450_m1). Assay mix was from Thermo Fisher Scientific. Realtime PCR (AbiPrism7500 Fast Sequence Detector, Thermo Fisher Scientific): two initial steps at 50°C (2 min) and 95°C (20 s) were followed by 40 cycles at 95°C (3 s) and 60°C (30 s). Detection of the dequenched probe, calculation of threshold cycles (*C*_T_ values), and data analysis were performed by the Sequence Detector software. Relative changes in mRNA expression compared with unstimulated control and normalized to GAPDH were quantified by the 2^−ΔΔCT^ method. As IFNα and IFNβ gene loci lack introns, all RNA isolates were digested with RNase-free DNase I (Thermo Fisher Scientific) before reverse transcription.

Interferonα was analyzed by standard PCR using universal primers that target all α-subtypes: forward, 5′-ATGGCTAGRCTCTGTGCTTTCCT-3′; revers, 5′-AGGGCTCTCCA GAYTTCTGCTCTG-3′. GAPDH: forward, 5′-CTGGCATTGCTCTCAATGAC-3′; revers, 5′-TCTTACTCCTTGGAGGCC-3′. PCR conditions: 95°C for 10 min (1 cycle), 95°C for 30 s, 62°C (IFNα) or 55°C (GAPDH) for 30 s, and 72°C for 45 s (with 37 cycles for IFNα and 25 cycles for GAPDH), and a final extension phase at 72°C for 7 min. Amplicon length: IFNα, 524nt; GAPDH, 110nt. Amplicons was confirmed by sequencing (Eurofins, Ebersberg, Germany).

### Chromatin Immunoprecipitation (ChIP)

Chromatin immunoprecipitation was performed as previously described (21). For immunoprecipitation, an IgG control or a specific STAT1 antibody was used (rabbit polyclonal antibody; Santa Cruz Biotechnology, Heidelberg, Germany). To amplify murine iNOS promoter regions enclosing relevant STAT1-binding sites [−951 to −912 bp relative to the transcriptional start site (TSS) (8)], the following primers were used for PCR (9): forward, 5′-ccaactattgaggccacacac-3′ (−1,098 to −1,078 bp); reverse, 5′-gcttccaat aaagcattcaca-3′ (−889 to −869 bp). Conditions: 95°C for 10 min (1 cycle), 95°C for 30 s, 56°C for 30 s, 72°C for 45 s (40 cycles), and final extension (72°C, 7 min). Amplicons were confirmed by sequencing (Eurofins).

### Murine Model of Experimental APAP-Induced Liver Injury

C57BL/6 mice were maintained under SPF conditions at the “Zentrale Tierhaltung” (Paul-Ehrlich-Institut, Langen, Germany). Type I IFN-receptor (IFNAR) chain 1-deficient mice, lacking a functional receptor for type I IFN (IFNAR^−/−^ mice), were approximately 20× backcrossed on the C57BL/6 background (22). All animal experiments using C57Bl/6 mice [male, 9–10-week old, wild-type (wt), and IFNAR^−/−^ mice] were carried out in accordance with the recommendations of the Animal Protection Agency of the Federal State of Hessen (Regierungspräsidium Darmstadt, Germany). The protocol was approved by the Regierungspräsidium Darmstadt (Germany).

Murine APAP (500 mg/kg)-induced liver injury was performed as described (23). Briefly, fasted male mice obtained i.p. injection of either warm 0.9% NaCl (B. Braun, Melsungen, Germany) or 500 mg/kg (dissolved in warm 0.9% NaCl) APAP. Mice that obtained NaCl are depicted as control mice (ctrl) throughout the manuscript. Mice had free access to food and water. After 6 h (only wt-mice) or 24 h (wt- and IFNAR^−/−^ mice), mice underwent isoflurane (Abbott, Wiesbaden, Germany) anesthesia and were sacrificed thereafter. Blood was taken from the retroorbital venous plexus. Serum alanine aminotransferase (ALT) activity was quantified according to manufacturer’s instructions (Reflotron, Roche Diagnostics, Mannheim, Germany). Serum was stored at −80°C. For RNA and protein analysis, liver tissue was snap frozen in liquid nitrogen and stored at −80°C. For histological analysis, liver tissue was perfused with PBS via the portal vein followed by overnight incubation in 4.5% buffered formalin. Thereafter, tissue was embedded in paraffin for histologic analysis. Paraffin-embedded liver sections (4 µm) were stained with hematoxylin (Applichem, Darmstadt, Germany). The degree of histopathological liver injury was quantified by Keyence BZ-II Analyzer software (Neu-Isenburg, Germany). Specifically, computer-aided analysis of tissue necrosis was performed by using similarly located liver sections obtained from 9 wt- and 9 IFNAR-deficient individual mice (*n* = 9 per genotype) treated with APAP (500 mg/kg, 24 h). One complete liver section per individual mice underwent analysis. The software quantifies the degree of liver necrosis by identifying necrotic areas based on differences in hematoxylin staining. Results are presented as text-only and expressed as (%-reduction) of liver necrosis observed in IFNAR-deficient mice compared with wt-mice.

### Immunohistochemical Detection of iNOS

Paraffin-embedded liver sections (4 µm) were used for detection of iNOS. Briefly, sections were deparaffinized and unmasked by heat treatment (Target Retrieval Solution; Dako, Glostrup, Denmark). Sections were stained using either a self-made in-house (24, 25) or a commercially available (Enzo Life Sciences, Lörrach, Germany) rabbit polyclonal antimurine iNOS antibody overnight at 4°C. Notably, both iNOS detecting antibodies generated analogous iNOS staining in livers of APAP-treated mice. For detection, goat antirabbit ABC staining system (Santa Cruz Biotechnology) and the 3,3′-diaminobenzidine Substrate Kit for Peroxidase (Sigma-Aldrich) were used. Sections were counterstained with hematoxylin.

### Immunoblot Analysis

Tissue homogenates were generated as previously described (23). Briefly, cells or liver homogenates were generated using lysis buffer [150 mM NaCl, 1 mM CaCl_2_, 25 mM Tris-Cl (pH 7.4), 1% Triton X-100], supplemented with protease inhibitor cocktail (Roche Diagnostics) and DTT, Na_3_VO_4_, PMSF (each 1 mM), and NaF (20 mM). Thereafter, SDS-PAGE and immunoblotting were performed. To detect iNOS or pSTAT1 together with GAPDH on the same blot, the blot was cut. Antibodies: iNOS, rabbit polyclonal antibody (Enzo Life Sciences); GAPDH, rabbit polyclonal antibody (Trevigen, Gaithersburg, USA); pSTAT1 (Tyr-701), rabbit polyclonal antibody (Cell Signaling, Frankfurt, Germany). Quantifications of immunoblots were performed by Quantity-One analysis software (Bio-Rad, Munich, Germany). As a “positive control” for iNOS expressing murine C57BL/6 tissue, cutaneous wound lysates obtained 3 days after skin wounding were used. At that time point iNOS protein expression in the wounded skin peaks (26). Wound lysates of iNOS-deficient mice from the same time point were analyzed as “negative control.” Those cutaneous wound lysates were kindly provided by Dr. Itamar Goren and Prof. Stefan Frank (University Hospital, Goethe University Frankfurt, *pharmazentrum frankfurt*). All animal experiments were approved by the Regierungspräsidium Darmstadt (Germany).

### Analysis of Nitrite Production

Griess assays (Merck, Darmstadt, Germany) were performed as described (10). Briefly, nitrite, a stable NO metabolite, was determined in cell-free supernatants using the Griess reagent (Merck, Darmstadt, Germany). Supernatants were mixed with equal volume of Griess reagent. The absorbance was measured at 540 nm using a microplate reader and nitrite concentrations were calculated using a sodium nitrite calibration curve.

### Statistics

Data were checked with the Kolmogorov–Smimov test for parametric distribution and are shown as mean ± SD (*in vitro*) or SEM (*in vivo*) (fold-induction or raw data relative to GAPDH, percent of input, Adj. Vol. INT*mm^2^, units/liter, or micromolar). Statistics was performed on raw data as indicated by either one-way analysis of variance with *post hoc* Bonferroni correction or by unpaired Student’s *t*-test (Prism 5.0, GraphPad, La Jolla, CA, USA).

## Results

### IFNβ Amplifies Hepatocyte iNOS Expression in Cell Culture

**Figure 1:**
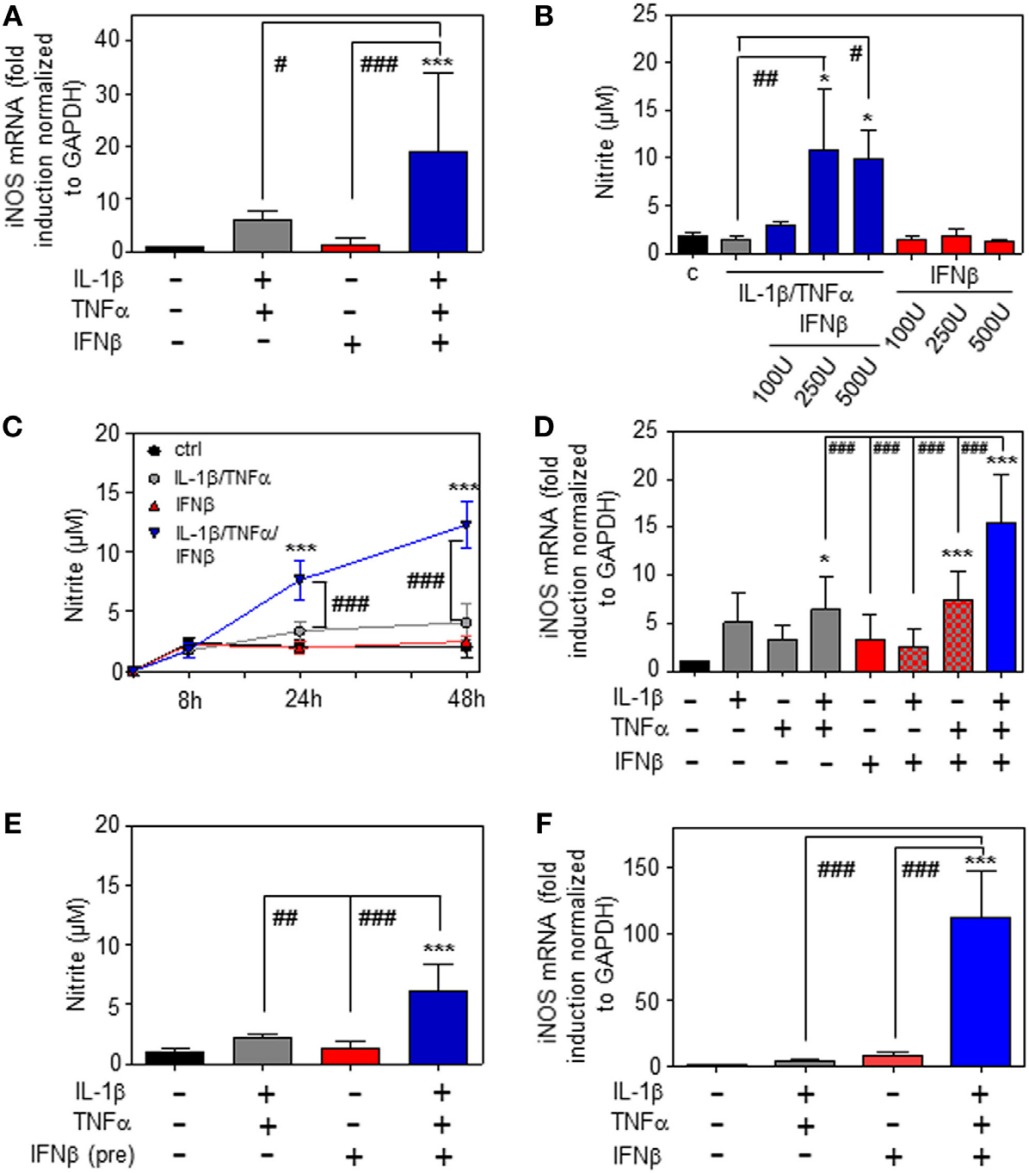
Inducible nitric oxide synthase (iNOS) upregulation by interferon (IFN)β in murine hepatoma cells. **(A–E)** Hepa1-6 cells were either kept as unstimulated control or stimulated with interleukin (IL)-1β (2 ng/ml), tumor necrosis factor (TNF)α (2 ng/ml), IL-1β/TNFα (each 2 ng/ml), IFNβ [at 250 U/ml **(A,C–E)** or the indicated concentrations **(B)**], or with IL-1β/TNFα (each 2 ng/ml)/IFNβ [at 250 U/ml **(A,C–E)** or the indicated concentrations **(B)**]. **(E)** Before addition of IL-1β/TNFα, cells were pre-incubated with IFNβ for 1 h. After 16 h **(A)**, 24 h **(B,E)**, 8 h **(D)**, or the indicated time periods **(C)** cells and culture supernatants were harvested. **(A,D)** iNOS mRNA determined by realtime PCR was normalized to that of GAPDH and is shown as fold-induction compared with unstimulated control [mean ± SD, *n* = 5 **(A)**, *n* = 4 **(D)**; *P < 0.05, ***P < 0.001 versus unstimulated control, *^#^P* < 0.05, ^###^*P* < 0.001]. **(B,C,E)** Nitrite production was determined using the Griess-assay [mean ± SD, *n* = 4; *P < 0.05, ***P < 0.001 versus unstimulated control (at the indicated time point **(C)**), ^#^*P* < 0.05, ^##^*P* < 0.01, ^###^*P* < 0.001]. **(F)** Hepa56.1D cells were either kept as unstimulated control or stimulated with IL-1β/TNFα (each 2 ng/ml), IFNβ (250 U/ml), or IL-1β/TNFα (each 2 ng/ml)/IFNβ (250 U/ml). After 16 h cells were harvested and mRNA determined by realtime PCR was normalized to that of GAPDH and is shown as fold-induction compared with unstimulated control (mean ± SD, *n* = 3; ***P < 0.001 versus unstimulated control, ^###^*P* < 0.001). **(A–F)** Statistical analysis, raw data were analyzed by one-way ANOVA with *post hoc* Bonferroni correction.

**Figure 2:**
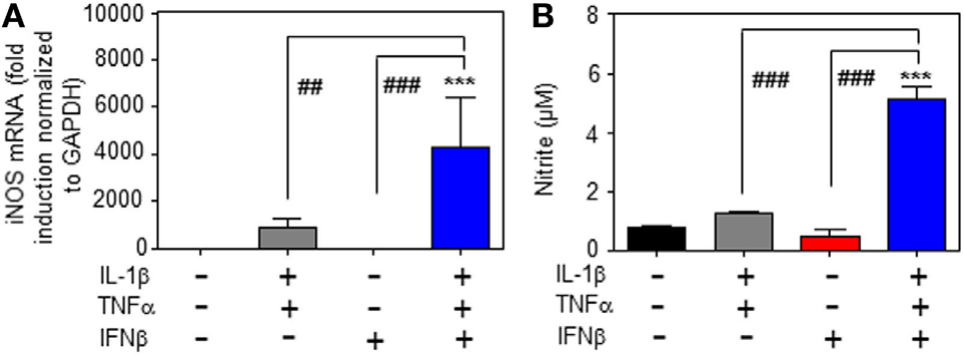
Inducible nitric oxide synthase (iNOS) upregulation by interferon (IFN)β in primary murine hepatocytes. **(A,B)** Primary murine hepatocytes were either kept as unstimulated control or stimulated with interleukin (IL)-1β/tumor necrosis factor (TNF)α (each 2 ng/ml), with IFNβ (at 250 U/ml), or with IL-1β/TNFα (each 2 ng/ml)/IFNβ (at 250 U/ml). After 8 h **(A)** and 16 h **(B)** cells and culture supernatants were harvested. **(A)** iNOS mRNA determined by realtime PCR was normalized to that of GAPDH and is shown as fold-induction compared with unstimulated control (mean ± SEM, *n* = 3; ***P < 0.001 versus unstimulated control, ^##^*P* < 0.01, ^###^*P* < 0.001). Statistical analysis, raw data were analyzed by one-way ANOVA with *post hoc* Bonferroni correction. **(B)** Nitrite production was determined using the Griess-assay (mean ± SEM, *n* = 3; ***P < 0.001 versus unstimulated control, ^###^*P* < 0.001). Statistical analysis, data were analyzed by one-way ANOVA with *post hoc* Bonferroni correction.

Whereas type I IFN is an established inducer of iNOS in monocytes/macrophages (9, 13–15), information on effects of IFNα/β on hepatocyte iNOS is scarce. Notably, one earlier report demonstrated that iNOS in human hepatoma Huh7 cells is not induced by IFNα as sole stimulus. Interactions with other cytokines were not investigated in that earlier report (27). To further investigate this matter, murine Hepa1-6 hepatoma cells were exposed to IFNβ in the presence or absence of NF-κB-activating cytokines IL-1β and TNFα (28). The combination IL-1β plus TNFα was employed since pilot experiments in Hepa1-6 cells (data not shown) demonstrated that this combination synergizes for induction of the prototypic hepatocyte-derived NF-κB-dependent chemokine MIP2 (29). Herein, we demonstrate that IFNβ in cooperation with aforementioned pro-inflammatory cytokines potentiates iNOS expression (Figure 1A) and activity as detected by nitrite release (Figure 1B). Potentiation of nitrite release by coincubation with IFNβ was stable over a 48 h time period (Figure 1C). Notably, in accord with the aforementioned report (27), IFNβ failed to induce iNOS as sole stimulus (Figures 1A–C). Detailed analysis furthermore revealed that triplet stimulation by IL-1β/TNFα/IFNβ is superior to that by either IL-1β/IFNβ or TNFα/IFNβ (Figure 1D) and that potentiation of iNOS is likewise detectable in the context of IFNβ preincubation (Figure 1E). Amplification of hepatocyte iNOS by IFNβ was not confined to Hepa1-6 cells. Synergism between IL-1β/TNFα and IFNβ for iNOS expression was actually even more pronounced in alternate murine Hepa56.1D hepatoma cells (Figure 1F). Moreover, IFNβ likewise potentiated iNOS in murine primary C57BL/6 hepatocytes which was readily detectable on mRNA (Figure 2A) and nitrite level (Figure 2B).

**Figure 3:**
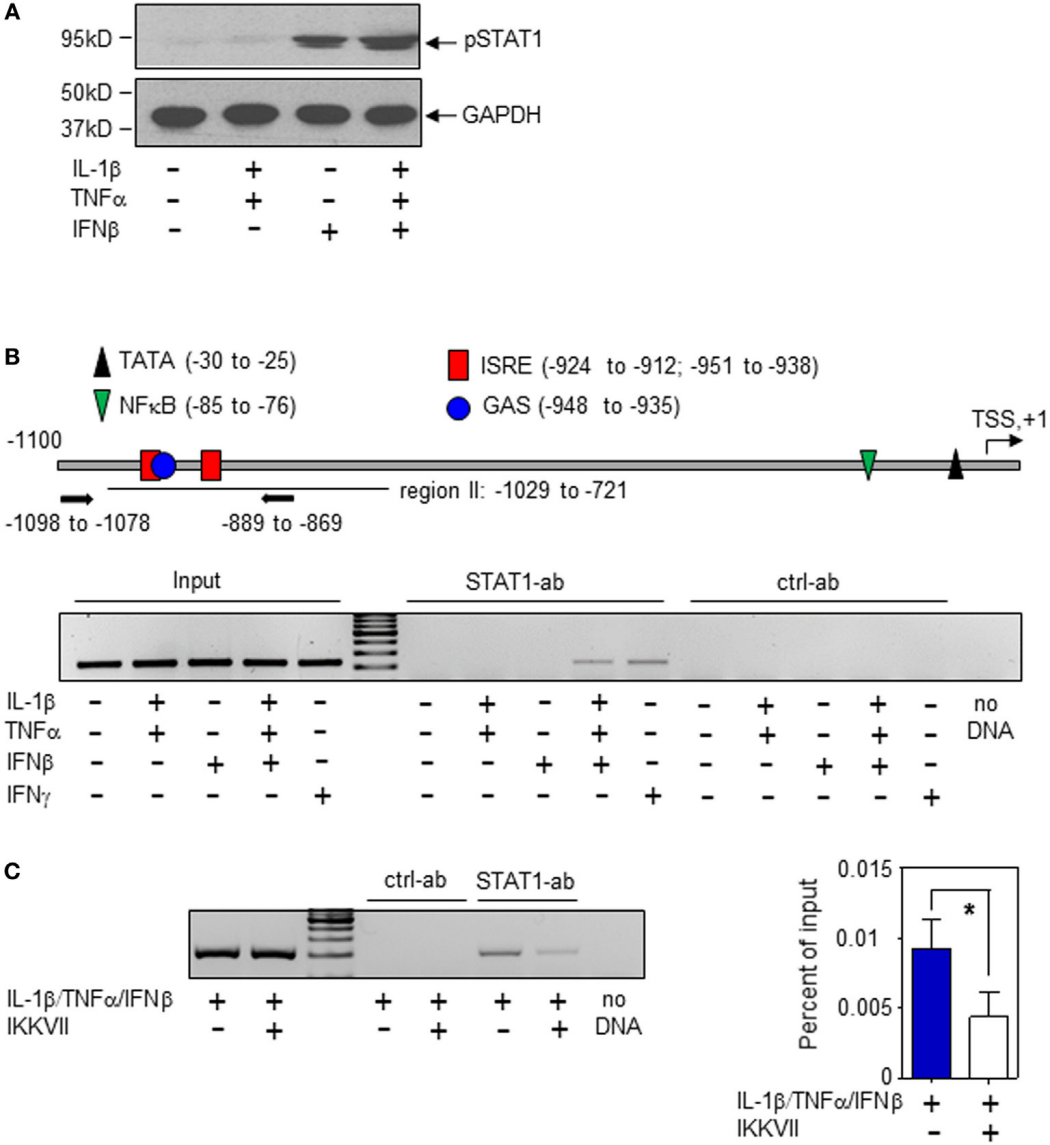
Interferon (IFN)β-induced signal transducer and activator of transcription (STAT)1 activation and inducible nitric oxide synthase (iNOS) expression in murine Hepa1-6 cells. **(A)** Cells were either kept as unstimulated control or stimulated with interleukin (IL)-1β/tumor necrosis factor (TNF)α (each 2 ng/ml), IFNβ (250 U/ml), or IL-1β/TNFα (each 2 ng/ml)/IFNβ (250 U/ml) for 1 h. pSTAT1 and GAPDH were determined by immunoblotting. One representative of three independently performed experiments is shown. [**(B)**, upper panel)] Schematic of the murine iNOS promoter (8). Critical regions of STAT1 (GAS/ISRE-sites) and nuclear factor (NF)-κB binding as well as the TATA-Box and the transcriptional start site (TSS) are indicated. Primers used for chromatin immunoprecipitation (ChIP)-PCR are depicted. [**(B)**, lower panel)] Cells were either kept as unstimulated control or stimulated with IL-1β/TNFα (each 2 ng/ml), IFNβ (250 U/ml), IL-1β/TNFα (each 2 ng/ml)/IFNβ (250 U/ml), or with IFNγ (10 ng/ml). After 2 h, ChIP analysis was performed for detection of STAT1 binding to the illustrated GAS/ISRE-promoter region (−1,098 to −869 bp). One representative of three independently performed experiments is shown. **(C)** Cells were either kept as unstimulated control or stimulated with IL-1β/TNFα (each 2 ng/ml)/IFNβ (250 U/ml). Where indicated, cells were pre-incubated with inhibitor-κB kinase-VII inhibitor (10 µM) for 30 min. After 2 h, ChIP analysis was performed for detection of STAT1 binding to the illustrated GAS/ISRE-promoter region. One representative of three independently performed experiments is shown (left panel). Right panel, densitometric quantification of the PCR signals from the left panel (*n* = 3; *P < 0.05). Statistical analysis, data were analyzed by Student’s *t*-test.

In order to characterize molecular mechanisms of murine hepatic iNOS gene induction by IFNβ, we chose to focus herein on Hepa1-6 hepatoma cells. Activation of the transcription factor STAT1 is key to immunoregulation by IFNα/β (30, 31). STAT1 activation, assessed by analysis of Tyr-701 phosphorylation (pSTAT1), was readily detectable in Hepa1-6 cells under the influence of IFNβ (Figure 3A). Within the murine iNOS promoter, a specific region (−912 to −1,029 bp relative to the TSS) was found to mediate STAT1-induced iNOS transcriptional activation which may be achieved by STAT1-homodimers (in response to IFNγ or type I IFN) or by the type I IFN/ISGF3 axis. In this region (Figure 3B, upper panel), adjacent STAT1-binding elements are located. Namely, a dual GAS/IFN-stimulated response element (ISRE) sequence (−951 to −935 bp)—binding STAT1 homodimers or ISGF3—and an additional ISRE site (−924 to −912 bp)—likewise potentially binding ISGF3 (8, 9, 31). STAT1 binding to this region was investigated herein to further characterize mechanisms mediating IFNβ potentiation of iNOS in Hepa1-6 hepatoma cells. In the presence of IL-1β/TNFα, ChIP analysis revealed STAT1 binding to this site in response to IFNβ which suggests enhanced STAT1-dependent transcriptional activity at the iNOS promoter. Unexpectedly, IFNβ as single stimulus failed to initiate STAT1 binding in Hepa1-6 cells. This was in contrast to IFNγ and may propose pivotal action of ISGF3 signaling in the context of stimulation by IFNβ. Data indicate that, in Hepa1-6 cells, signaling by IL-1β/TNFα enforces IFNβ-induced STAT1 binding to the iNOS promoter thereby enabling synergistic gene induction. As expected, IL-1β/TNFα-stimulation, without IFNβ, did not mediate STAT1 binding to this promoter region (Figure 3B, lower panel). In order to further deepen the connection between IL-1β/TNFα and IFNβ-related STAT1 binding to the hepatocyte iNOS promoter, NF-κB activation was inhibited by exposing cells to the IKK inhibitor IKK-VII (32). As detected by ChIP analysis, STAT1 binding to the aforementioned region of the iNOS promoter (−912 to −1,029 bp relative to the TSS) was significantly impaired under the influence of IKK-VII (Figure 3C). Data indicate that NF-κB activation by IL-1β/TNFα, likely mediated by an active proximal NF-κB site at −85 to −76 bp relative to the TSS (8), supports STAT1 binding to the iNOS promoter in murine Hepa1-6 hepatoma cells.

### Type I IFN Supports Expression of iNOS during APAP-Induced Liver Injury

To investigate regulation of hepatic iNOS by type I IFN *in vivo*, the model of moderate APAP-induced acute liver inflammation was used. Liver injury and thus associated necroinflammation in this model is at its peak at around 24 h after APAP administration. Notably, 48 h after onset of intoxication serum ALT levels, indicative of liver necrosis, drop to approximately or below 20% of those detectable at 24 h with liver morphology displaying regeneration and recovery from injury (33–38). In light of these characteristics, we chose to focus herein on the 24 h time point after APAP administration. Notably, this APAP toxicity is associated with a cytokine response that includes upregulation of IL-1β and TNFα (23, 39). Previous reports demonstrated that, during murine APAP intoxication, iNOS protein is well detectable (33, 40) in hepatocytes at regions with centrilobular injury (41, 42). Evaluation of iNOS knockout mice indicated that iNOS-derived NO may promote injury during early intoxication (detected by serum ALT) (43, 44). In contrast, hepatotoxicity was found to be independent from iNOS analyzed histochemically after 24 h. The role of iNOS in APAP-induced liver injury appears complex since NO also inhibits generation of poisonous *N*-acetyl-p-benzoquinone imine from APAP and reduces superoxide anion-dependent lipid peroxidation (43).

**Figure 4:**
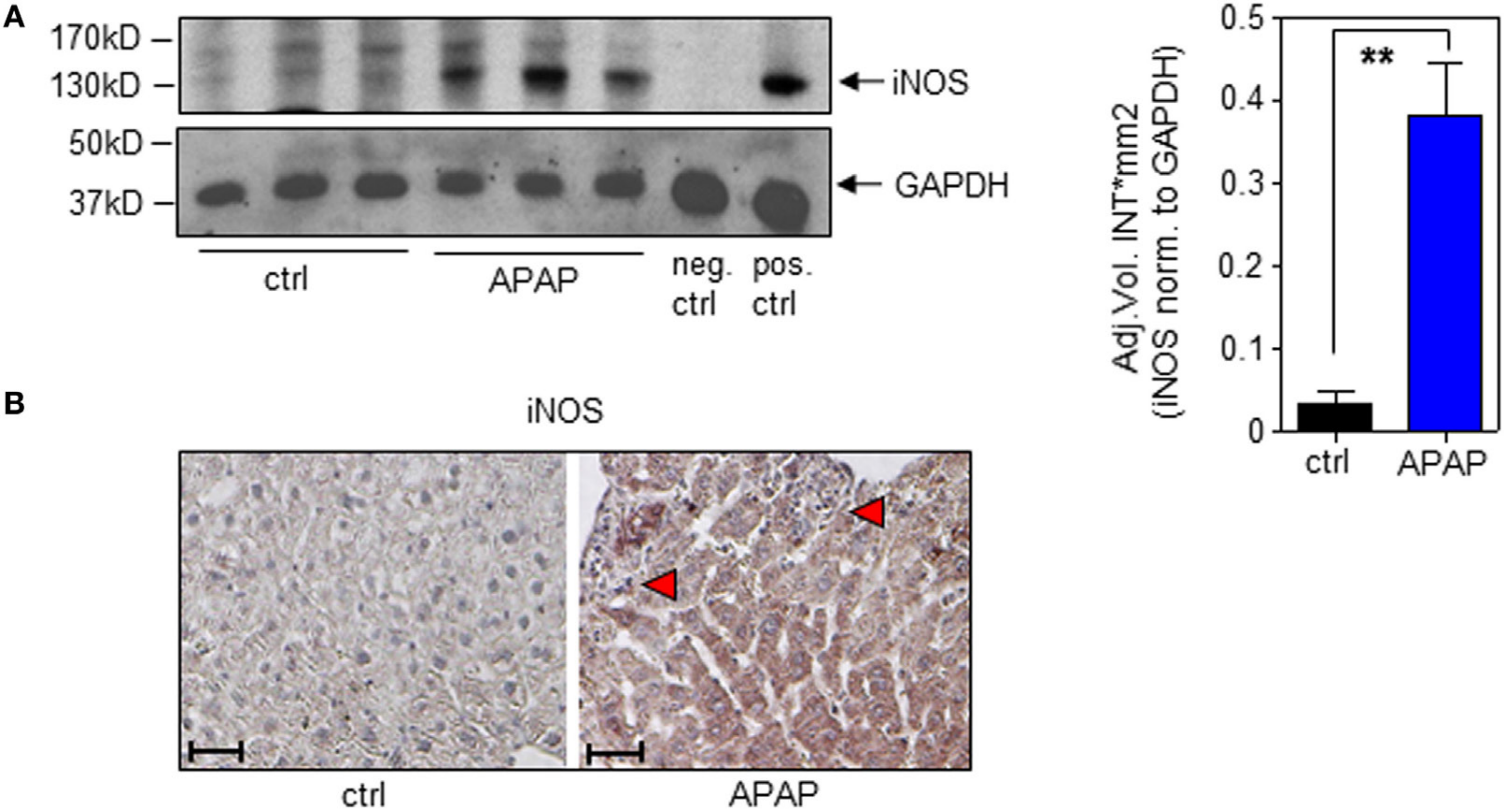
Hepatic inducible nitric oxide synthase (iNOS) expression in acetaminophen (APAP)-induced liver injury. Wild-type mice received either NaCl-ctrl solution or APAP (500 mg/kg) and were maintained for 24 h. **(A)** Left panel, hepatic iNOS was determined by immunoblotting. Right panel, densitometric quantification of iNOS from left panel (mean ± SEM, *n* = 3 individual mice per group; **P < 0.01). Statistics, Student’s *t*-test. pos.-ctrl., iNOS-positive murine 3-day-after-wounding skin; neg.-ctrl., murine 3-day-after-wounding skin from iNOS^−/−^ mice (see Materials and Methods). **(B)** Paraffin sections were stained immunohistologically for iNOS with hematoxylin counterstaining. Shown are representative liver sections of four control specimens and six specimens obtained from APAP-treated mice. Antibody, rabbit polyclonal antimurine iNOS antibody (Enzo Life Sciences); magnification, 10×; scale, 100 µm; red triangles indicate areas of necrosis.

In the present study, we confirm increased hepatic iNOS protein in APAP-treated mice (Figure 4A; with densitometric quantification, right panel). Immunohistochemistry likewise corroborated iNOS protein expression by hepatocytes during APAP intoxication which was absent in ctrl-mice (Figure 4B).

**Figure 5:**
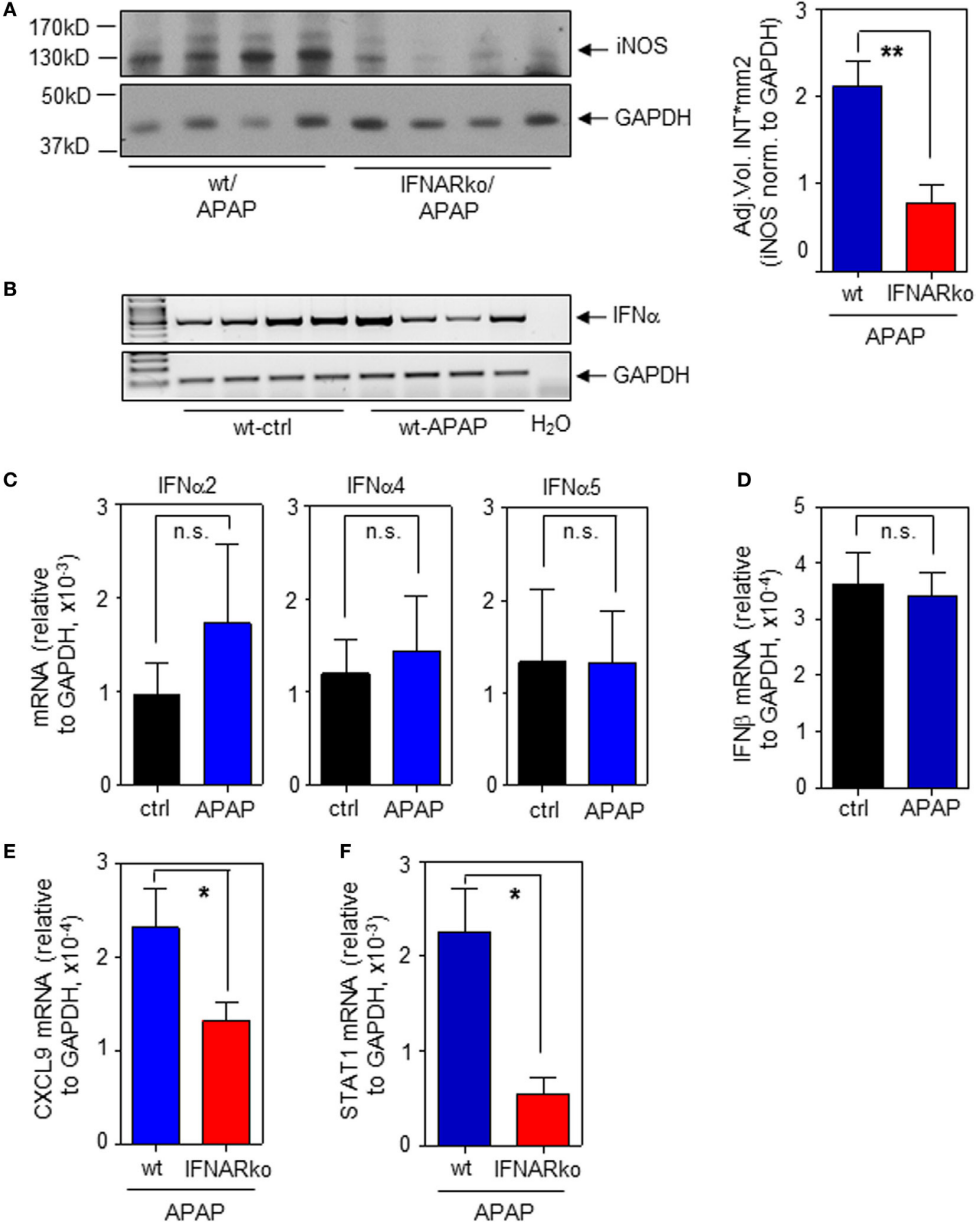
Impaired hepatic inducible nitric oxide synthase (iNOS) expression in interferon-receptor (IFNAR)^−/−^ mice during acetaminophen (APAP)-induced liver injury. Wild-type (wt) and IFNAR^−/−^ mice received NaCl-ctrl solution or APAP (500 mg/kg) and were maintained for 24 h. **(A)** Left panel, hepatic iNOS was determined by immunoblotting (shown are four individual mice per genotype). Right panel, densitometric quantification of iNOS from the left panel with two additional mice per genotype (mean ± SEM, *n* = 6; **P < 0.01). **(B)** Hepatic IFNα mRNA was determined by standard PCR using universal primers that target all α-subtypes (*n* = 4 individual mice per group). **(C)** Hepatic IFNα2 (left panel), IFNα4 (middle panel), and IFNα5 (right panel) mRNA were determined by realtime PCR and normalized to GAPDH (mean ± SEM; all subtypes, *n* = 4). **(D)** Hepatic IFNβ was determined by realtime PCR and normalized to GAPDH (mean ± SEM; ctrl, *n* = 4; APAP, *n* = 5). **(E)** Hepatic CXCL9 mRNA was determined by realtime PCR and normalized to GAPDH (mean ± SEM; wt, *n* = 8 individual mice; IFNAR^−/−^, *n* = 9; *P < 0.05). **(F)** Hepatic STAT1 mRNA was determined by realtime PCR and normalized to GAPDH (mean ± SEM; *n* = 9 individual mice per genotype; *P < 0.05). **(A,E,F)** Statistical analysis, raw data were analyzed by Student’s *t*-test.

To determine the relevance of type I IFN for iNOS expression in the context of APAP-induced liver injury, experiments were performed by using IFNAR^−/−^ mice. Those mice are unable to respond to type I IFN (22, 31). In fact, induction of hepatic iNOS protein was impaired in IFNAR-deficient mice (Figure 5A; with densitometric quantification, right panel). In a next step, expression of hepatic IFNα/β was determined in order to further assess type I IFN action during APAP intoxication. Notably, we did not observe upregulation of hepatic IFNα/β mRNA as detected 6 h (data not shown) or 24 h after administration of APAP. However, basal constitutive type I IFN was readily detectable in all liver specimens investigated. Figure 5B demonstrates constitutive hepatic IFNα expression as detected by standard PCR using primers targeting the entire panel of murine α-subtypes. Those results were confirmed by realtime PCR for detection of IFNα2 (Figure 5C, left panel), IFNα4 (Figure 5C, middle panel), and IFNα5 (Figure 5C, right panel). Likewise, hepatic IFNβ was expressed constitutively (Figure 5D).

Besides iNOS, IFNAR-deficient mice exposed to APAP likewise displayed decreased hepatic mRNA of STAT1-dependent CXCL9 (Figure 5E). We and others have previously reported that STAT1 gene expression is regulated by IFN/STAT1-driven positive feedback regulation (16, 31, 45–47). Accordingly, decreased hepatic STAT1 mRNA was likewise detected in IFNAR-deficient mice (Figure 5F) which likely contributes to downregulation of STAT1-inducible genes such as iNOS.

**Figure 6:**
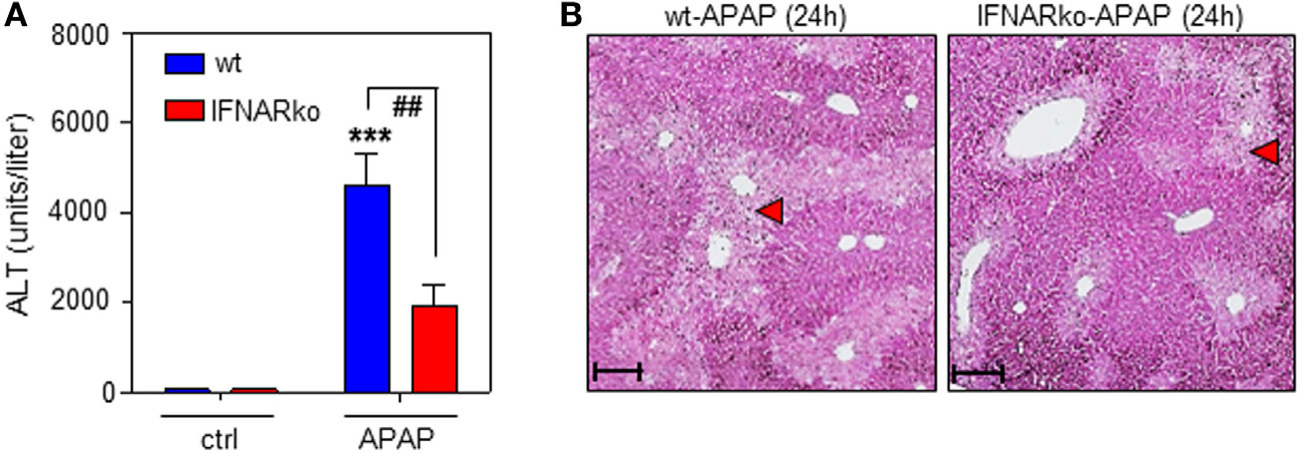
Acetaminophen (APAP)-induced liver injury in wild-type (wt) and interferon-receptor (IFNAR)^−/−^ mice during APAP-induced liver injury. **(A,B)** wt or IFNAR^−/−^ mice received NaCl-ctrl solution (*n* = 4 individual mice per genotype) or APAP (500 mg/kg; *n* = 9 individual mice per genotype). **(A)** After 24 h, serum ALT was determined and is depicted as units/liter (mean ± SEM; ***P < 0.001 versus NaCl-ctrl solution-treated mice of the same genotype, ^##^*P* < 0.01). Statistical analysis, raw data were analyzed one-way ANOVA with *post hoc* Bonferroni correction. **(B)** Representative liver sections (H&E stain) 24 h after the onset of APAP intoxication. 10×; scale, 200 µm; red triangles indicate areas of necrosis.

Finally, the role of IFNAR concerning the severity of APAP-induced liver injury was assessed. This issue is in fact controversially discussed. Whereas a previous study reported no effects of IFNAR deficiency on intoxication (35), another study observed pathological action of type I IFN. Specifically, administration of IFNAR-neutralizing antibodies diminishes murine APAP-induced liver damage. Moreover, intoxication is enhanced in genetically engineered mice displaying impaired IFNAR degradation but ameliorated when degradation is enforced by pharmacological means (48). In support of this latter view, herein, reduced APAP toxicity connected to IFNAR deficiency which was observed by analysis of serum ALT (Figure 6A) and histological software-aided evaluation 24 h after administration of 500 mg/kg APAP (29.6 ± 5.3% reduction of liver necrosis in IFNAR-deficient versus wt mice; *n* = 9, *P* < 0.01 by unpaired Student’s *t*-test). Figure 6B displays histochemistry of representative APAP-induced hepatic injury in wt and IFNAR^−/−^ mice, respectively.

## Discussion

Type I IFN is a key cytokine component of innate immunity supposed to affect course of disease particularly in viral but also during bacterial infections and, due to a substantial immunoregulatory potential, likewise in sterile inflammation (30, 49–52).

Herein, type I IFN is characterized *in vitro* and *in vivo* as significant determinant of hepatic iNOS expression having the potential to determine disease outcome during liver inflammation. On a cellular level, we demonstrate that IFNβ potently synergizes with the prototypic inflammatory cytokines IL-1β/TNFα for induction of iNOS in primary murine hepatocytes and murine Hepa56.1D as well as Hepa1-6 hepatoma cells. As determined in this latter cellular model, IFNβ directs STAT1 binding to a critical regulatory site within the murine iNOS promoter (−912 to −1,029 bp relative to the TSS) (8, 9), a process that, in Hepa1-6 hepatoma cells, demanded simultaneous pro-inflammatory signaling by IL-1β/TNFα. Notably, a recent report identified in murine bone marrow-derived macrophages (BMDM) an additional distal STAT1 binding region 30 kB upstream of the iNOS TSS which may be able to regulate gene expression (53). However, using IL-1β/TNFα/IFNβ-stimulated Hepa1-6 cells we confirm evident binding of STAT1 to the aforementioned proximal promoter region. Combined with previous studies (8, 9), present data thus emphasize the relevance of this proximal STAT1-binding region for iNOS induction. Interestingly, IFNγ but not IFNβ as single stimulus mediated STAT1 binding to this site—suggesting a role for ISGF3. In contrast to the present observations using Hepa1-6 cells, IFNβ as single stimulus induces STAT1 binding to this proximal binding region in BMDM (9) which indicates cell type-specific mechanisms at work. The unexpected requirement of IL-1β/TNFα signaling for STAT1 binding to the iNOS promoter observed herein is a further cell-type specific facet of the well-described STAT1/NF-κB synergism that drives inflammatory/antimicrobial gene expression (53). Regulation of CXCL9 in murine NIH3T3 fibroblasts may serve as a leading case in that context. There, efficient STAT1 binding to the CXCL9 promoter demands TNFα costimulation and downstream STAT1/NF-κB bridging by CREB binding protein—though STAT1 homodimers are involved in this case (54). The notion of cooperative transcription factor binding, specifically NF-κB enforcing STAT1 binding to the iNOS promoter in IL-1β/TNFα/IFNβ-stimulated Hepa1-6 cells, was confirmed herein by pharmacological inhibition of NF-κB.

Upregulation of iNOS *in vivo* during murine APAP-induced liver injury and sterile inflammation in fact largely depended on type I IFN signaling which associated with pronounced liver injury. Whereas hepatic type I IFN, as assessed by analysis of IFNα/β expression, was not upregulated during APAP intoxication, basal type I IFN was well detectable in murine liver tissue, an observation that agrees with previous reports on constitutive murine hepatic IFNα (55) and IFNβ (56), respectively. Notably, the liver is regarded a major target for constitutively produced type I IFN in healthy mice (57) and low-level “physiological” expression of type I IFN also applies to human liver tissue and hepatocytes (58, 59). By generally promoting signal transduction mechanisms related to cellular activation, constitutive low-level expression of type I IFN is supposed to prime diverse tissues for immunological alertness (45) which may in particular apply to the liver as crucial host/environment-interface serving “firewall” functions (60).

It must, however, be emphasized that regulatory properties of type I IFN during hepatic inflammation are multilayered and context dependent. This is exemplified by the general ability of type I and II IFN to potently upregulate anti-inflammatory IL-1 receptor antagonist (IL-1Ra) (61) which, by inhibiting IL-1 biological activity, enables protection in murine models of nucleic acid (virus)-induced liver damage (22, 62). Interestingly, the role of IL-1 in APAP-induced liver injury is actually discussed controversially with disease aggravating action (63, 64), no significant role (65), or even protective functions (66) being ascribed to this cytokine. Of note, administration of IL-1Ra did not affect disease in the current protocol of APAP-induced liver injury (Bachmann and Mühl, unpublished data), an observation supporting aforementioned previous report (65). Data thus indicate that putative upregulation of potentially protective IL-1Ra by surplus type I IFN falls short in the current pathophysiological context.

Data presented not only relate to sterile inflammation as seen in APAP intoxication but likewise connect to infectious diseases such as viral hepatitis. Interestingly, hepatocytes express iNOS protein during chronic hepatitis C virus (HCV) infection (67) which, according to data presented herein, should be supported by induction of endogenous type I IFN in response to the virus (68). Moreover, HCV patients responding most efficiently to IFNα therapy likewise display increased serum nitrite/nitrate levels (69), an established surrogate marker of iNOS activation during infectious diseases (70).

Taken together, current knowledge and data presented herein suggest that IFNβ supports hepatocyte iNOS by dual complementary action. That is, IFN signaling directly triggers STAT1 biological activity, a process further enhanced by feed-forward upregulation of STAT1 gene expression. Data also suggest a pathogenic role for constitutive type I IFN during the course of APAP intoxication which is regarded a prototypic model for drug-induced injury and sterile inflammation at the liver compartment.

## Ethics Statement

All animal experiments using C57Bl/6 mice (male, 9–10-week-old, wt, and IFNAR^−/−^ mice) were carried out in accordance with the recommendations of the Animal Protection Agency of the Federal State of Hessen (Regierungspräsidium Darmstadt, Germany). The protocol was approved by the Regierungspräsidium Darmstadt (Germany).

## Author Contributions

HM analyzed the data, designed the study, wrote the paper, and performed manuscript editing. MB performed all experiments, analyzed the data, and contributed to manuscript writing and editing. ZW analyzed the data, provided mice, and technical support. TP provided crucial technical support. JP analyzed the data, provided reagents (antibodies), and contributed to manuscript editing.

## Conflict of Interest Statement

The authors declare that the research was conducted in the absence of any commercial or financial relationships that could be construed as a potential conflict of interest.
